# Bayesian inference for psychology. Part I: Theoretical advantages and practical ramifications

**DOI:** 10.3758/s13423-017-1343-3

**Published:** 2017-08-04

**Authors:** Eric-Jan Wagenmakers, Maarten Marsman, Tahira Jamil, Alexander Ly, Josine Verhagen, Jonathon Love, Ravi Selker, Quentin F. Gronau, Martin Šmíra, Sacha Epskamp, Dora Matzke, Jeffrey N. Rouder, Richard D. Morey

**Affiliations:** 1 Department of Psychological Methods, University of Amsterdam, Nieuwe Achtergracht 129-B, 1018 VZ Amsterdam, The Netherlands; 2 Masaryk University, Brno, Czech Republic; 3 University of Missouri, Columbia, MO USA; 4 Cardiff University, Cardiff, UK

**Keywords:** Hypothesis test, Statistical evidence, Bayes factor, Posterior distribution

## Abstract

Bayesian parameter estimation and Bayesian hypothesis testing present attractive alternatives to classical inference using confidence intervals and *p* values. In part I of this series we outline ten prominent advantages of the Bayesian approach. Many of these advantages translate to concrete opportunities for pragmatic researchers. For instance, Bayesian hypothesis testing allows researchers to quantify evidence and monitor its progression as data come in, without needing to know the intention with which the data were collected. We end by countering several objections to Bayesian hypothesis testing. Part II of this series discusses JASP, a free and open source software program that makes it easy to conduct Bayesian estimation and testing for a range of popular statistical scenarios (Wagenmakers et al. this issue).

*Theoretical satisfaction and practical implementation are the twin ideals of coherent statistics.* Dennis Lindley, 1980.

The psychology literature is rife with *p* values. In almost every published research article in psychology, substantive claims are supported by *p* values, preferably ones smaller than .05. For instance, the December 2014 issue of *Psychonomic Bulletin & Review* featured 24 empirical brief reports, all of which reported *p* values. The dominance of the *p* value statistical framework is so complete that its presence feels almost prescriptive (“every empirical article in psychology shall feature at least one *p* value.”). In Part I of this two-part series we aim to demonstrate that there exists a valid and feasible alternative –Bayesian inference– whose adoption brings considerable benefits, both in theory and in practice.

Based on a superficial assessment, the continued popularity of *p* values over Bayesian methods may be difficult to understand. The concept of *p* value null hypothesis statistical testing (NHST) has been repeatedly critiqued on a number of important points (e.g., Edwards, Lindman, & Savage, 1963; Morrison & Henkel, 1970; Mulaik & Steiger, 1997; Wagenmakers, 2007), and few methodologists have sought to defend the practice. One of the critiques is that *p* values are often misinterpreted as Bayesian posterior probabilities, such that it is all too easy to believe that *p* < .05 warrants the rejection of the null hypothesis $\mathcal {H}_{0}$, and consequently supports the acceptance of the alternative hypothesis $\mathcal {H}_{1}$. This interpretation of *p* values is tempting but incorrect (Gigerenzer, Krauss, & Vitouch, 2004). A *p* value is the probability of obtaining results at least as extreme as those observed given that the null hypothesis is true. The transition from this concept to the decision, “I accept the alternative hypothesis”, is a leap that is logically invalid. The *p* value does not take into account the prior plausibility of $\mathcal {H}_{0}$, and neither does it recognize the fact that data unusual under $\mathcal {H}_{0}$ can also be unusual under $\mathcal {H}_{1}$ (Wagenmakers et al. in press). Other pressing problems with *p* values will be discussed shortly.

From a psychological perspective, however, a number of arguments may help explain the continued popularity of *p* values over Bayesian methods.^1^ First, researchers practice and preach the methodology that they were once taught themselves; interrupting this self-perpetuating educational cycle requires that researchers invest serious effort to learn new methods. Second, by breaking away from the dominant group of *p* value practitioners, researchers choose to move away from the in-group and expose themselves to the associated risks of academic exclusion. Third, just like fish form schools to escape predation, researchers may believe that there is security in repeating procedures that are popular; “surely,” they may feel, “if the procedure I use is standard in the field, then any detractors must be overstating their case”. Fourth, many psychologists are primarily interested in addressing substantive research questions, not in the finer details of statistical methodology; such methodological disinterest feeds the desire for simple procedures that work well enough to convince the reviewers. In this sense the current *p* value fixation is similar to a statistical ritual (i.e., the “null ritual”, Gigerenzer, 2004). Fifth, the *p* value framework, when misinterpreted, offers a simple solution to deal with the uncertainty inherent in noisy data: when *p* < .05, reject $\mathcal {H}_{0}$ and accept $\mathcal {H}_{1}$; when *p* > .10, retain $\mathcal {H}_{0}$. When misapplied in this way, *p* values appear to make it easy for researcher to draw strong conclusions even when the empirical results are noisy and uninformative. Sixth, researchers may feel that by using non-standard methods (i.e., anything other than the *p* value) they reduce their chances of getting their work published or having it understood by their colleagues. Seventh, researchers interested in methodology have often internalized their statistical education to such an extent that they have difficulty accepting that the method they have used all their life may have serious limitations; when new information conflicts with old habits, the resulting cognitive dissonance can be reduced by discounting or ignoring the new information. Finally, it is possible that researchers may agree with the *p* value critiques, yet are unable to adopt alternative (Bayesian) inferential procedures. The reason for this inability is straightforward: virtually all statistical software packages produce *p* values easily, whereas Bayesian methods cannot count on the same level of support. Many of these arguments hold for statistical innovations in general, not just for *p* value NHST (Sharpe 2013).

In general, then, powerful psychological and societal forces are at play, making it nigh impossible to challenge the dominant methodology. Nonetheless, the edifice of NHST appears to show subtle signs of decay. This is arguably due to the recent trials and tribulations collectively known as the “crisis of confidence” in psychological research, and indeed, in empirical research more generally (e.g., Begley & Ellis, 2012; Button et al., 2013; Ioannidis, 2005; John, Loewenstein, & Prelec, 2012; Nosek & Bar-Anan, 2012; Nosek, Spies, & Motyl, 2012; Pashler & Wagenmakers, 2012; Simmons, Nelson, & Simonsohn, 2011). This crisis of confidence has stimulated a methodological reorientation away from the current practice of *p* value NHST. A series of recent articles have stressed the limitations of *p* values and proposed alternative methods of analysis (e.g., Cumming, 2008, 2014; Halsey, Curran-Everett, Vowler, & Drummond, 2015; Johnson, 2013; Kruschke, 2010a, 2011; Nuzzo, 2014; Simonsohn, 2015b). In response, flagship journals such as *Psychological Science* have issued editorials warning against the uncritical and exclusive use of *p* values (Lindsay 2015); similar warnings have been presented in the *Psychonomic Bulletin & Review* Statistical Guidelines for authors; finally, the journal *Basic And Applied Social Psychology* has banned *p* values altogether (Trafimow & Marks 2015).

In order to reduce psychologists’ dependence on *p* values it is essential to present alternatives that are concrete and practical. One such alternative is inference from confidence intervals (i.e., the “new statistics”, Cumming, 2014; Grant, 1962). We see two main limitations for the new statistics. The first limitation is that confidence intervals are not Bayesian, which means that they forego the benefits that come with the Bayesian approach (a list of such benefits is provided below); moreover, confidence intervals share the fate of *p* values in the sense that they are prone to fallacies and misinterpretations (Greenland et al., in press; Morey, Hoekstra, Rouder, Lee, & Wagenmakers, 2016). The second limitation is that confidence intervals presume that the effect under consideration exists; in other words, their use implies that every problem of inference is a problem of parameter estimation rather than hypothesis testing. Although we believe that effect size estimation is important and should receive attention, the question of size (“how big is the effect?”) comes into play only after the question of presence (“is there an effect?”) has been convincingly addressed (Morey, Rouder, Verhagen, & Wagenmakers, 2014). In his monograph “Theory of Probability”, Bayesian pioneer Harold Jeffreys makes a sharp distinction between estimation and testing, discussing each in separate chapters: “In the problems of the last two chapters we were concerned with the estimation of the parameters in a law, the form of the law itself being given. We are now concerned with the more difficult question: in what circumstances do observations support a change of the form of the law itself? *This question is really logically prior to the estimation of the parameters, since the estimation problem presupposes that the parameters are relevant.*” (Jeffreys, 1961, p. 245; italics ours). The same sentiment was recently expressed by Simonsohn (2015b, p. 559): “Only once we are past asking whether a phenomenon exists at all and we come to accept it as qualitatively correct may we become concerned with estimating its magnitude more precisely. Before lines of inquiry arrive at the privileged position of having identified a phenomenon that is generally accepted as qualitatively correct, researchers require tools to help them distinguish between those that are and are not likely to get there.” We believe it is a mistake to mandate either an estimation or a testing approach across the board; instead, the most productive mode of inference depends on the substantive questions that researchers wish to have answered. As illustrated below, the problems with *p* values are not a reason to abandon hypothesis testing – they are a reason to abandon *p* values.

As a concrete and practical alternative to hypothesis testing using *p* values, we propose to conduct hypothesis testing using Bayes factors (e.g., Berger, 2006; Jeffreys, 1935, 1961; Kass & Raftery, 1995). The Bayes factor hypothesis test compares the predictive adequacy of two competing statistical models, thereby grading the evidence provided by the data on a continuous scale, and quantifying the change in belief that the data bring about for the two models under consideration. Bayes factors have many practical advantages; for instance, they allow researchers to quantify evidence, and they allow this evidence to be monitored continually, as data accumulate, and without needing to know the intention with which the data were collected (Rouder 2014; Wagenmakers 2007).

In order to profit from the practical advantages that Bayesian parameter estimation and Bayes factor hypothesis tests have to offer it is vital that the procedures of interest can be executed in accessible, user-friendly software package. In part II of this series (Wagenmakers et al. this issue) we introduce JASP (https://jasp-stats.org/; JASP Team, 2016), a free and open-source program with a graphical user interface familiar to users of SPSS. With JASP, users are able to conduct classical analyses as well as Bayesian analyses, without having to engage in computer programming or mathematical derivation.

The overarching goal of Part I this series is to present Bayesian inference as an attractive alternative to *p* value NHST. To this end, a concrete example is used to highlight ten practical advantages of Bayesian parameter estimation and Bayesian hypothesis testing over their classical counterparts. Next we briefly address a series of ten objections against the Bayes factor hypothesis test. Our hope is that by raising awareness about Bayesian benefits (and by simultaneously providing a user-friendly software program, see Wagenmakers et al., this issue) we can help accelerate the adoption of Bayesian statistics in psychology and other disciplines.

## Bayesian inference and its benefits

**Fig. 1:**
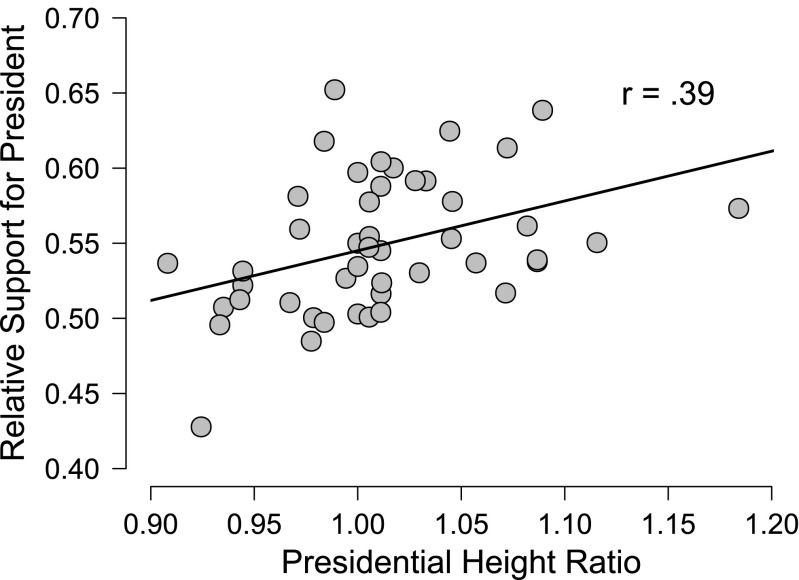
The proportion of the popular vote versus the height ratio between a US president and his closest competitor for the first 46 elections. Data obtained from Stulp et al. (2013). Figure based on JASP

To facilitate the exposition below we focus on a concrete example: the height advantage of candidates for the US presidency (Stulp, Buunk, Verhulst, & Pollet, 2013). The data from the first 46 US presidential elections can be analyzed in multiple ways, but here we are concerned with the Pearson correlation *ρ* between the proportion of the popular vote and the height ratio (i.e., height of the president divided by the height of his closest competitor). Figure 1 shows that taller candidates tend to attract more votes; the sample correlation *r* equals .39 and is significantly different from zero (*p* = .007, two-sided test). A classical confidence interval for *ρ* ranges from .12 to .61. We now turn to a Bayesian analysis of these data, first discussing estimation, then discussing hypothesis testing of the correlation *ρ*. Our exposition is necessarily brief and selective; a complete treatment of Bayesian inference requires a monograph (e.g., Bernardo & Smith, 1994; Jeffreys, 1961; Jaynes, 2003; Lunn, Jackson, Best, Thomas, & Spiegelhalter, 2012; O’Hagan & Forster, 2004). In addition, we have made an effort to communicate the concepts and ideas without recourse to equations and derivations. Readers interested in the mathematical underpinnings of Bayesian inference are advised to turn to other sources (e.g., Ly, Verhagen, & Wagenmakers, 2016b; Marin & Robert, 2007; O’Hagan & Forster, 2004; Pratt et al., 1995; Rouder et al., 2012; an overview and a reading list are provided in this issue, Etz, Gronau, Dablander, Edelsbrunner, & Baribault, this issue).

### Bayesian Parameter Estimation

**Fig. 2:**
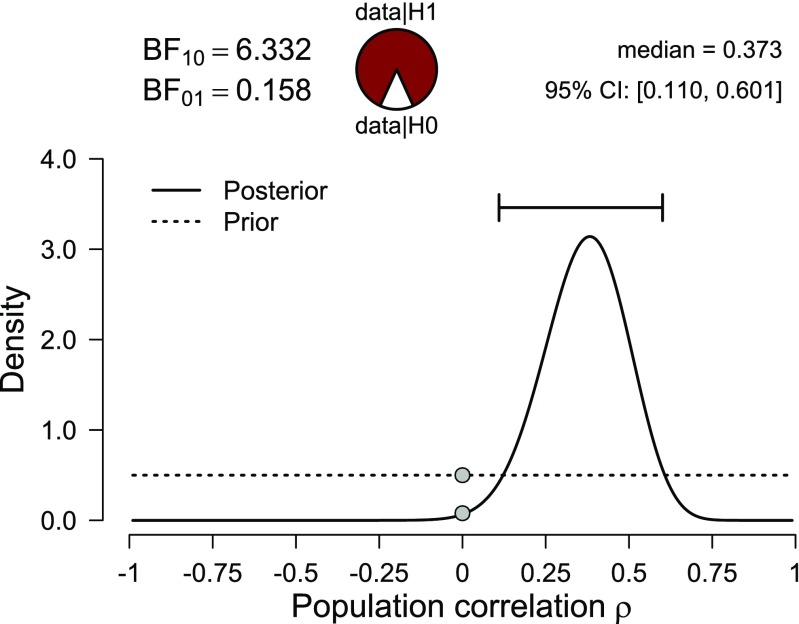
Prior and posterior distribution for the correlation between the proportion of the popular vote and the height ratio between a US president and his closest competitor. The default two-sided Bayes factor is visualized by the ratio between the prior and posterior ordinate at *ρ* = 0 and equals 6.33 in favor of the alternative hypothesis over the null hypothesis. Figure from JASP

A Bayesian analysis may proceed as follows. The model under consideration assumes that the data are bivariate Normal, and interest centers on the unknown correlation coefficient *ρ*. In Bayesian statistics, the uncertainty about *ρ* before seeing the data is quantified by a probability distribution known as the prior. Here we specify a default prior distribution, one that stipulates that every value of *ρ* is equally plausible a priori (Jeffreys 1961); this yields a uniform distribution ranging from − 1 to 1, shown in Fig. 2 by the dotted line.^2^ It is possible to specify different models by changing the prior distribution. For instance, later we will incorporate the knowledge that *ρ* is expected to be positive, which can be accomplished by using a uniform prior distribution that ranges only from 0 to 1. For the moment, we refrain from doing so here because the classical NHST analysis is also two-sided.

Next the prior distribution is combined with the information from the data (i.e., the likelihood; Edwards, 1992; Myung, 2003; Royall, 1997) and the result is a posterior distribution. This posterior distribution quantifies the uncertainty about *ρ* after having seen the data. Figure 2 shows that compared to the prior distribution, the posterior distribution assigns relatively little mass to values lower than 0 and higher than .70. A 95% credible interval ranges from .11 to .60, which means that one can be 95% confident that the true value of *ρ* lies between .11 and .60. When the posterior distribution is relatively peaked compared to the prior, this means that the data were informative and much has been learned. Note that the area under the prior and the posterior distribution has to equal 1; consequently, if some values of *ρ* are less likely under the posterior then they were under the prior, the reverse pattern needs to hold for at least some other values of *ρ*.

### Benefits of Bayesian parameter estimation

In psychology, Bayesian parameter estimation techniques have recently been promoted by Jeff Rouder and colleagues (e.g., Rouder, Lu, Speckman, Sun, & Jiang, 2005; Rouder, Lu, et al., 2007; Rouder, Lu, Morey, Sun, & Speckman, 2008), by Michael Lee and colleagues (e.g., Lee, 2008, 2011; Lee, Fuss, & Navarro, 2006), and by John Kruschke (e.g., Kruschke, 2010a, 2010b, 2011). Because the results of classical parameter estimation techniques (i.e., confidence intervals) are sometimes numerically similar to those obtained using Bayesian methods (i.e., credible intervals), it is tempting to conclude that the difference is not of practical interest. This is, however, a misconception. Below we indicate several arguments in favor of Bayesian parameter estimation using posterior distributions over classical parameter estimation using confidence intervals. For more details and examples see Morey et al. (2016). Before proceeding, it is important to recall the definition of a classical confidence interval: An *X* *%* confidence interval for a parameter *𝜃* is an interval generated by a procedure that in repeated sampling has an *X* *%* probability of containing the true value of *𝜃* (Hoekstra, Morey, Rouder, & Wagenmakers, 2014; Neyman, 1937). Thus, the confidence in the classical confidence interval resides in its performance in repeated use, across hypothetical replications. In contrast, the confidence in the Bayesian credible interval refers directly to the situation at hand (see benefit 3 below and see Wagenmakers, Morey, & Lee, 2016). Table 1 lists five benefits of Bayesian estimation over classical estimation. We will discuss each in turn.

**Table 1:** Select overview of advantages of Bayesian inference over classical inference

	Bayesian Inference	Classical Inference	References
Desiderata for Parameter Estimation			
1. To incorporate prior knowledge			1,2
2. To quantify confidence that *𝜃* lies in a specific interval			3
3. To condition on what is known (i.e., the data)			4,5
4. To be coherent (i.e., not internally inconsistent)			6,7
5. To extend naturally to complicated models			8,9
Desiderata for Hypothesis Testing			
1. To quantify evidence that the data provide for $\mathcal {H}_{0}$ vs. $\mathcal {H}_{1}$			10,11
2. To quantify evidence in favor of $\mathcal {H}_{0}$			12,13
3. To allow evidence to be monitored as data accumulate			14,15
4. To not depend on unknown or absent sampling plans			16,17
5. To not be “violently biased” against $\mathcal {H}_{0}$			18,19,20

See text for details. References: 1 = Dienes (2011); 2 = Vanpaemel (2010); 3 = (Pratt et al. 1995, p. 258); 4 = Berger & Wolpert (1988); 5 = Jaynes (2003); 6 = Lindley (1985); 7 = Lindley (2000); 8 = Pratte & Rouder (2012); 9 = Lunn et al. (2012); 10 = Jeffreys (1935); 11 = Jeffreys (1961); 12 = Rouder et al. (2009); 13 = Wagenmakers (2007); 14 = Edwards et al. (1963); 15 = Rouder (2014); 16 = Berger & Berry (1988); 17 = Lindley (1993); 18 = W. Edwards (1965); 19 = Berger & Delampady (1987); 20 = Sellke et al. (2001)

#### Benefit 1. Bayesian estimation can incorporate prior knowledge

The posterior distribution is a compromise between the prior (i.e., what was known before the data arrived), and the likelihood (i.e., the extent to which the data update the prior). By selecting an appropriate prior distribution, researchers are able to insert substantive knowledge and add useful constraint (Vanpaemel 2010; Vanpaemel & Lee 2012). This is not a frivolous exercise that can be misused to obtain arbitrary results (Lindley 2004). For instance, consider the estimation of IQ. Based on existing knowledge, it is advisable to use a Gaussian prior distribution with mean 100 and standard deviation 15. Another example concerns the estimation of a participant’s latent ability to discriminate signal from noise in a psychophysical present-absent task. In the absence of ability, the participant still has a 50% probability of guessing the correct answer. Hence, the latent rate *𝜃* of correct judgements is bounded from below by 0.5 (Morey, Rouder, & Speckman, 2008; Rouder, Morey, Speckman, & Pratte, 2007). Any statistical paradigm that cannot incorporate such knowledge seems overly restrictive and incomplete. The founding fathers of classical inference –including “Student” and Fisher– mentioned explicitly that their methods apply only in the absence of any prior knowledge (Jeffreys 1961, pp. 380–382).

**Fig. 3:**
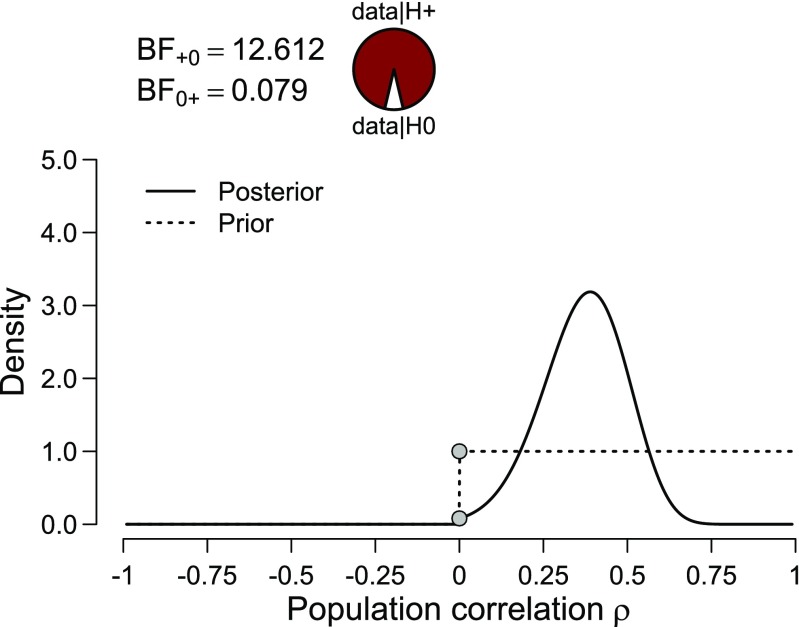
One-sided prior and posterior distribution for the correlation between the proportion of the popular vote and the height ratio between a US president and his closest competitor. The default one-sided Bayes factor is visualized by the ratio between the prior and posterior ordinate at *ρ* = 0 and equals 12.61 in favor of the alternative hypothesis over the null hypothesis. Figure from JASP

To see how easy it is to add meaningful constraints to the prior distribution, consider again the example on the US presidents (see also Lee & Wagenmakers, 2013; Wagenmakers, Verhagen, & Ly, 2016). Assume that, before the data were examined, the correlation was believed to be positive; that is, it was thought that taller candidates attract more votes, not less. This restriction can be incorporated by assigning *ρ* a uniform distribution from 0 to 1 (Hoijtink, Klugkist, & Boelen, 2008; Hoijtink, 2011; Klugkist, Laudy, & Hoijtink, 2005). The results are shown in Fig. 3. Note that the area under the one-sided prior distribution needs to equal 1, which explains why it is twice as high as the two-sided prior distribution shown in Fig. 2.

A comparison between Figs. 2 and 3 also reveals that the restriction did not meaningfully alter the posterior distribution. This occurs because most of the posterior mass was already consistent with the restriction, and hence the one-sided restriction necessitated only a minor adjustment to the posterior obtained from the two-sided prior. In contrast, the classical one-sided 95% confidence interval ranges from .16 to 1, containing all values that would not be rejected by a one-sided *α* = .05 significance test. This one-sided interval is very different from the two-sided interval that ranged from .12 to .61. In light of the data, and in light of the posterior distribution, the one-sided confidence interval does not appear to provide an intuitive or desirable summary of the uncertainty in estimating *ρ*.^3^ To further stress the difference between the Bayesian and classical one-sided intervals, note that for the present data the one-sided classical interval that presumes the opposite restriction (i.e., taller candidates are assumed to attract fewer votes) yields an interval that ranges from − 1 to 0.58, that is, covering all of the negative range and most of the positive range. In sharp contrast, the restriction to negative correlations yields a Bayesian one-sided credible interval with negative bounds that are very close to zero, as one would expect.

In sum, Bayesian estimation methods allow researchers to add substantive prior knowledge. The classical framework is incapable of doing so except for the simplest case of an order-restriction, where it yields intervals that do not provide useful information about the precision with which parameters were estimated.

#### Benefit 2. Bayesian estimation can quantify confidence that *𝜃* lies in a specific interval

The posterior distribution for a parameter *𝜃* provides a complete summary of what we know about this parameter. Using this posterior distribution, we can answer questions such as “how much more likely is the value *𝜃* = .6 versus the value *𝜃* = .4?” – this equals the ratio of the heights of the posterior distribution at those values. Also, we can use the posterior distribution to quantify how likely it is that *𝜃* falls in a specific interval, say, between .2 and .4 – this equals the posterior mass in that interval (Wagenmakers, Morey, & Lee, 2016).

In contrast, the classical confidence interval procedure can do no more than provide X% confidence intervals. It is not possible within the classical framework to specify the interval bounds and then ask for the probability or confidence that the true value is within these bounds. This is aserious limitation. For instance, one criterion for the diagnosis of an intellectual disability is an IQ below 70. Hence it may be important to know the probability that aperson’s IQ is in the interval from 0to 70, given aseries of test scores. With classical statistics, this question cannot be addressed. Pratt et al. (1995, p. 258) formulate this concern as follows:

A feature of confidence regions which is particularly disturbing is the fact that the confidence level must be selected in advance and the region we then look at is imposed by chance and may not be at all one we are interested in. Imagine the plight of amanager who exclaims, ‘I understand [does he?] the meaning that the demand for XYZ will lie in the interval 973 to 1374 with confidence .90. However, Iam particularly interested in the interval 1300 to 1500. What confidence can Iplace on that interval?’ Unfortunately, this question *cannot* be answered. Of course, however, it is possible to give aposterior probability to that particular interval—or any other—based on the sample data and on acodification of the manager’s prior judgments.

Cox (1958, p. 363) expresses asimilar concern (see also Lindley, 1965, p. 23):

(...) the method of confidence intervals, as usually formulated, gives only one interval at some preselected level of probability. (...) For when we write down the confidence interval (...) for acompletely unknown normal mean, there is certainly asense in which the unknown mean *𝜃* is likely to lie near the centre of the interval, and rather unlikely to lie near the ends and in which, in this case, even if *𝜃* does lie outside the interval, it is probably not far outside. The usual theory of confidence intervals gives no direct expression of these facts.

#### Benefit 3. Bayesian estimation conditions on what is known (i.e., the data)

The Bayesian credible interval (and Bayesian inference in general) conditions on all that is known. This means that inference is based on the specific data set under consideration, and that performance of the methodology for other hypothetical data sets is irrelevant. In contrast, the classical confidence interval is based on average performance across hypothetical data sets. To appreciate the difference, consider a scale that works perfectly in 95% of the cases, but returns a value of “1 kilo” in the remaining 5%. Suppose you weigh yourself on this scale and the result is “70 kg”. Classically, your confidence in this value should be 95%, because the scale is accurate in 95% of all cases. However, the data tell you that the scale has not malfunctioned, and hence you can be 100% confident in the result. Similarly, suppose the scale returns “1 kilo”. Classically, you can have 95% confidence in this result. Logically, however, the value of “1 kilo” tells you that the scale has malfunctioned, and you have learned nothing at all about your weight (Berger & Wolpert 1988).

Another example is the 50% confidence interval for a binomial rate parameter *𝜃* (i.e., *𝜃* is allowed to take on values between 0 and 1). A classically valid 50% interval can be constructed by ignoring the data and randomly reporting either the interval (0 − 0.5) or (0.5 − 1). This random interval procedure will cover the true value in 50% of the cases. Of course, when the data are composed of 10 successes out of 10 trials the interval (0 − 0.5) is nonsensical; however, the confidence of the classical procedure is based on average performance, and the average performance of the random interval is 50%.

**Fig. 4:**
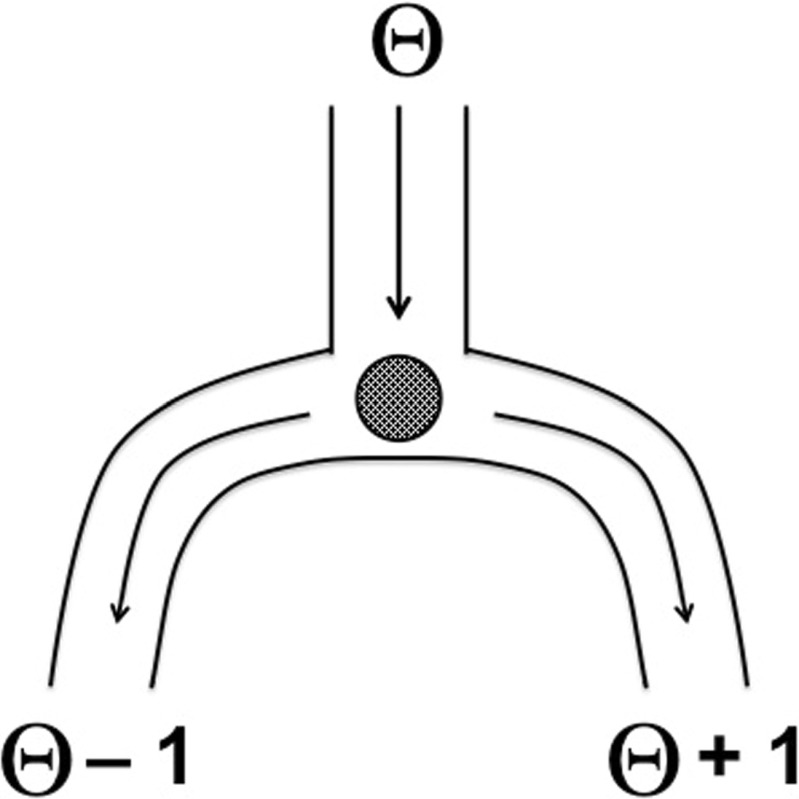
Two balls are dropped consecutively in a tube at location *𝜃*; each ball lands randomly at tube location *𝜃* − 1 or *𝜃* + 1. When the two balls land in different locations, *𝜃* is known with 100% certainty; when the two balls land in the same location, *𝜃* is known with 50% certainty. The pre-data average of 75% confidence is meaningless after the data have been observed. The example is taken from Berger & Wolpert (1988)

Thus, one of the crucial differences between classical and Bayesian procedures is that classical procedures are generally “pre-data”, whereas Bayesian procedures are “post-data” (Jaynes 2003).^4^ One final example, taken from by Berger & Wolpert (1988), should suffice to make the distinction clear. The situation is visualized in Fig. 4: two balls are dropped, one by one, in the central tube located at *𝜃*. Each ball travels down the central tube until it arrives at the T-junction, where it takes either the left or the right tube with equal probability, where the final outcome is registered as *𝜃* − 1 and *𝜃* + 1, respectively.

Consider that the first ball registers as “12”. Now there are two scenarios, both equally likely a priori, that provide radically different information. In the first scenario, the second ball lands in the other tube. For instance, the second ball can register as a “14”. In this case, we know with 100% certainty that *𝜃* is 13 – the middle value. In the second scenario, the second ball lands in the same tube as the first one, registering another “12”. This datum is wholly uninformative, as we still do not know whether *𝜃* equals 13 (when “12” is the left tube) or 11 (when “12” is the right tube). Hence we simply guess that the balls have traveled down the left tube and state that *𝜃* equals 13. The first scenario always yields 100% accuracy and the second scenario yields 50% accuracy. Both scenarios are equally likely to occur and hence the overall probability that the above procedure correctly infers the true value of *𝜃* is 75%. This indicates how well the procedure performs in repeated use, averaged across the sample space (i.e., all possible data sets).

However, consider that two balls have been observed and you are asked what you have learned about *𝜃*. Even classical statisticians agree that in cases such as these, one should not report an unconditional confidence of 75%; instead, one should take into account that the first scenario is different from the second, and draw different conclusions depending on the data at hand. As a technical side note, the negative consequences of averaging across hypothetical data sets that are fundamentally different is known as the problem of “recognizable/relevant subsets”. Ultimately, the problem can only be overcome by conditioning on the data that were observed, but doing so removes the conceptual basis of classical inference. In Bayesian inference, the problem of relevant subsets does not occur (for a more detailed discussion see e.g., Brown, 1967; Cornfield, 1969; Gleser, 2002; Morey et al., 2016; Pierce, 1973; Pratt, 1961). Relevant subsets are easy to detect in somewhat contrived examples such as the above; however, they also exist in standard inference situations such as the comparison of two means (Buehler & Fedderson 1963).

The conceptual and practical difference between classical and Bayesian intervals is eloquently summarized by Jaynes (1976, pp. 200–201):

Our job is not to follow blindly arule which would prove correct 90% of the time in the long run; there are an infinite number of radically different rules, all with this property. Our job is to draw the conclusions that are most likely to be right in the specific case at hand (...) To put it differently, the sampling distribution of an estimator is not ameasure of its reliability in the individual case, because considerations about samples that have not been observed, are simply not relevant to the problem of how we should reason from the one that has been observed. Adoctor trying to diagnose the cause of Mr. Smith’s stomachache would not be helped by statistics about the number of patients who complain instead of asore arm or stiff neck. This does not mean that there are no connections at all between individual case and long-run performance; for if we have found the procedure which is ‘best’ in each individual case, it is hard to see how it could fail to be ‘best’ also in the long run (...) The point is that the converse does not hold; having found arule whose long-run performance is proved to be as good as can be obtained, it does not follow that this rule is necessarily the best in any particular individual case. One can trade off increased reliability for one class of samples against decreased reliability or another, in away that has no effect on long-run performance; but has avery large effect on performance in the individual case.

#### Benefit 4. Bayesian estimation is coherent (i.e., not internally inconsistent)

One of the defining characteristics of Bayesian inference is that it is coherent, meaning that all inferential statements must be mutually consistent; in other words, Bayesian inference does not depend on the way aproblem is framed (de Finetti, 1974; Lindley, 1985, 2006; Ramsey, 1926). In Bayesian statistics, coherence is guaranteed by the laws of probability theory: “Coherence acts like geometry in the measurement of distance; it forces several measurements to obey the system.” (Lindley 2000, p. 306). For instance, when we know that for aposterior distribution, *p*(0 < *ρ* < 0.3) = *a* and *p*(0.3 < *ρ* < 0.4) = *b*, then it has to follow that *p*(0 < *ρ* < 0.4) = *a* + *b*. Any other conclusion violates the laws of probability theory and is termed incoherent or absurd (Lindley 1985). Afamous example of incoherence is provided by (Tversky & Kahneman 1983, p. 297), who gave participants the following background story:

Linda is 31 years old, single, outspoken and very bright. She majored in philosophy. As astudent, she was deeply concerned with issues of discrimination and social justice, and also participated in anti-nuclear demonstrations.

After reading the story, participants were asked to provide the probability of several statements, including the following two:

1. “Linda is a bank teller. (T)”
2. “Linda is a bank teller and is active in the feminist movement. (T&F)”

The results showed that the great majority of participants judged the conjunction statement T&F to be more probable than the constituent statement T. This conjunction error violates the laws of probability theory, according to which the probability of T&F can never be higher than the probability of either of its constituents (see also Nilsson, Winman, Juslin, & Hansson, 2009). Within the restrictions of the normative Bayesian framework, violations of logic and common sense can never occur.

Coherence is about fitting together different pieces of information in a way that is internally consistent, and this can be done in only one way: by obeying the laws of probability theory. Consider the following example. A bent coin is tossed twice: the first toss comes up heads, and the second toss comes up tails. Assume that, conditional on the angle of the bent coin, the tosses are independent. Then the final inference about the angle should not depend on the order with the data were observed (i.e., heads-tails or tails-heads). Similarly, the final inference should not depend on whether the data were analyzed sequentially, one at a time, or as a single batch. This sequential form of coherence can only be obtained by continual updating of the prior distribution, such that the posterior distribution after datum *i* becomes the prior distribution for the analysis of datum *i* + 1; without a prior distribution, coherence is impossible and inferential statements are said to be absurd. Coherence also ensures that Bayesian inference is equally valid for all sample sizes – there is no need for “rules of thumb” to identify sample sizes below which inference cannot be trusted.

Coherence has been argued to be the core element of Bayesian inference; for instance, Ramsey (1926) argued that “the most generally accepted parts of logic, namely, formal logic, mathematics and the calculus of probabilities, are all concerned simply to ensure that our beliefs are not self-contradictory” (see Eagle, 2011, p. 65); Jeffreys (1961, p. ix) starts the preface to the Bayesian classic “Theory of Probability” by stating that “The chief object of this work is to provide a method of drawing inferences from observational data that will be self-consistent and can also be used in practice”. Moreover, Lindley (1985) used the term “coherent statistics” instead of “Bayesian statistics”, and Joyce (1998) highlighted the importance of coherence by proving that “any system of degrees of belief that violates the axioms of probability can be replaced by an alternative system that obeys the axioms and yet is more accurate *in every possible world*” (see Eagle, 2011, p. 89).

In contrast to Bayesian inference, the concept of coherence plays no role in the classical framework. The resulting problems become manifest when different sources of information need to be combined. In the classical framework, the usual remedy against incoherence is to focus on one source of information only. Even though this hides the problem from view, it does not eliminate it, because almost any data set can be divided into arbitrary batches, and the final inference should not depend on the order or method of division.

#### Benefit 5. Bayesian estimation extends naturally to complicated models

The principles of Bayesian estimation hold for simple models just as they do for complicated models (e.g., Gelman & Hilll, 2007; Gelman et al., 2014). Regardless of model complexity, Bayesian inference features only one estimator: the posterior distribution. When this posterior distribution cannot be obtained analytically, it is usually possible to draw samples from it using numerical algorithms such as Markov chain Monte Carlo (MCMC; Gelfand & Smith, 1990; Gilks, Richardson, & Spiegelhalter, 1996; van Ravenzwaaij, Cassey, & Brown, in press). By increasing the number of MCMC samples, the posterior distribution can be approximated to arbitrary precision. With the help of MCMC sampling, Bayesian inference proceeds almost mechanically, allowing for straightforward inference even in relatively complex models (e.g., Lunn et al., 2012).

Consider the use of hierarchical nonlinear process models in cognitive psychology. Most models in cognitive psychology are *nonlinear* in that they are more than the sum of effects plus noise. An example of a nonlinear model is Yonelinas’ dual process model, in which memory performance is a mixture of recollection, modeled as a discrete all-or-none process, and familiarity, modeled as a continuous signal-detection process (e.g., Yonelinas, 2002). In realistic settings each of several people observe each of several items, but each person-item combination is unique. It is reasonable to assume variation across people and items, and once the model is expanded to include people and item effects, it is not only nonlinear, but quite numerous in parameters. One approach is to aggregate data across people, items, or both. The drawback is that the fit to aggregated data will be substantially distorted and perhaps reflect the psychological processing of nobody (Estes, 1956; Heathcote, Brown, & Mewhort, 2000; Rouder et al., 2005). A superior approach is to construct hierarchical nonlinear process models that simultaneously account for psychological process and nuisance variation from people and items. Pratte & Rouder (2012), for example, fit an expanded, hierarchical dual process model with about 2000 parameters. It is not obvious to us how to fit such models in a classical framework.^5^ Fortunately, the analysis is tractable and relatively straightforward using Bayesian inference with MCMC sampling.

Thus, Bayesian estimation is ideally suited for models that respect the complexity inherent in psychological data; such realistic models can be hierarchical, involve mixtures, contain nonlinearities, or be based on detailed considerations of the underlying psychological process (Lee & Wagenmakers, 2013; Shiffrin, Lee, Kim, & Wagenmakers, 2008). Despite their surface differences, all such models obey the same conceptual principles, and parameter estimation is merely amatter of “turning the Bayesian handle”:

“What is the principal distinction between Bayesian and classical statistics? It is that Bayesian statistics is fundamentally boring. There is so little to do: just specify the model and the prior, and turn the Bayesian handle. There is no room for clever tricks or an alphabetic cornucopia of definitions and optimality criteria. Ihave heard people who should know better use this dullness as an argument against Bayesianism. One might as well complain that Newton’s dynamics, being based on three simple laws of motion and one of gravitation, is apoor substitute for the richness of Ptolemys epicyclic system.” (Dawid 2000, p. 326)

### Bayesian hypothesis testing

In Bayesian parameter estimation, the inferential end-goal is the posterior distribution. In the earlier example featuring election outcomes, the posterior distribution for *ρ* allowed an answer to the question “What do we know about the correlation between height and popularity in the US elections, assuming from the outset that such a correlation exists?” From this formulation, it is clear that we cannot use the posterior distribution alone for the purpose of hypothesis testing: the prior formulation *ρ* ∼Uniform[−1,1] presupposes that *ρ* is relevant, that is, it presupposes that *ρ* is unequal to zero.^6^ To test an invariance or a general law, this law needs to be assigned a separate prior probability (Etz and Wagenmakers, 2016; Haldane, 1932; Jeffreys, 1961, 1973, 1980; Ly et al., 2016b; Wrinch & Jeffreys, 1921, 1923): to test $\mathcal {H}_{0}: \rho = 0$, this hypothesis needs to be taken serious a priori. In the election example, this means that we should explicitly consider the hypothesis that taller candidates do not attract a larger or smaller proportion of the popular vote. This is something that the estimation framework fails to do. Consequently, as stated by Berger (2006, p. 383): “[...] Bayesians cannot test precise hypotheses using confidence intervals. In classical statistics one frequently sees testing done by forming a confidence region for the parameter, and then rejecting a null value of the parameter if it does not lie in the confidence region. This is simply wrong if done in a Bayesian formulation (and if the null value of the parameter is believable as a hypothesis).”

Hence, when the goal is hypothesis testing, Bayesians need to go beyond the posterior distribution. To answer the question “To what extent do the data support the presence of a correlation?” one needs to compare two models: a null hypothesis that states the absence of the effect (i.e., $\mathcal {H}_{0}: \rho = 0$) and an alternative hypothesis that states its presence. In Bayesian statistics, this alternative hypothesis needs to be specified exactly. In our election scenario, the alternative hypothesis we discuss first is specified as $\mathcal {H}_{1}: \rho \sim \text {Uniform}(-1,1)$, that is, every value of *ρ* is judged to be equally likely a priori (Jeffreys 1961; Ly et al. 2016b).^7^

With the competing hypotheses $\mathcal {H}_{0}$ and $\mathcal {H}_{1}$ fully specified, the process of updating their relative plausibilities is described by a simplification of Bayes’ rule:

1 $$ \underbrace{\frac{p(\mathcal{H}_{1} \mid \text{data})}{p(\mathcal{H}_{0} \mid \text{data})}}_{\text{Posterior odds}}=\underbrace{\frac{p(\mathcal{H}_{1})}{p(\mathcal{H}_{0})}}_{\text{Prior odds}} \times \,\, \underbrace{\frac{p(\text{data} \mid \mathcal{H}_{1})}{p(\text{data} \mid \mathcal{H}_{0})}}_{\text{Bayes factor BF}_{10}}.  $$

In this equation, the prior model odds $p(\mathcal {H}_{1})/p(\mathcal {H}_{0})$ indicate the relative plausibility of the two models before seeing the data. After observing the data, the relative plausibility is quantified by the posterior model odds, that is, $p(\mathcal {H}_{1} \mid \text {data}) / p(\mathcal {H}_{0} \mid \text {data})$. The change from prior to posterior odds brought about by the data is referred to as the Bayes factor, that is, $p(\text {data} \mid \mathcal {H}_{1}) / p(\text {data} \mid \mathcal {H}_{0})$. Because of the subjective nature of the prior model odds, the emphasis of Bayesian hypothesis testing is on the amount by which the data shift one’s beliefs, that is, on the Bayes factor. When the Bayes factor BF_10_ equals 6.33, the data are 6.33 times more likely under $\mathcal {H}_{1}$ than under $\mathcal {H}_{0}$. When the Bayes factor equals BF_10_ = 0.2, the data are 5 times more likely under $\mathcal {H}_{0}$ than under $\mathcal {H}_{1}$. Note that the subscripts “10” in BF_10_ indicate that $\mathcal {H}_{1}$ is in the numerator of Eq. 1 and $\mathcal {H}_{0}$ is in the denominator, whereas the subscripts “01” indicate the reverse. Hence, BF_10_ = 1/BF_01_.

An alternative interpretation of the Bayes factor is in terms of the models’ relative predictive performance (Wagenmakers, Grünwald, & Steyvers, 2006; Wagenmakers, Morey, & Lee, 2016). Consider two models, $\mathcal {H}_{0}$ and $\mathcal {H}_{1}$, and two observations, *y* = (*y*_1_,*y*_2_). The Bayes factor BF_10_(*y*) is given by $p(y_{1},y_2 \mid \mathcal {H}_{1}) / p(y_{1},y_2 \mid \mathcal {H}_{0})$, that is, the ratio of the advance probability that the competing models assign to the data. Thus, both models make a probabilistic prediction about the data, and the model with the best prediction is preferred. This predictive interpretation can also be given a sequential slant. To see this, recall that according to the definition of conditional probability, *p*(*y*_1_,*y*_2_) = *p*(*y*_1_)*p*(*y*_2_∣*y*_1_). In the current example, both $\mathcal {H}_{0}$ and $\mathcal {H}_{1}$ make a prediction about the first data point, yielding $\text {BF}_{10}(y_{1}) = p(y_{1} \mid \mathcal {H}_{1}) / p(y_{1} \mid \mathcal {H}_{0})$ – the relative predictive performance for the first data point. Next, both models incorporate the knowledge gained from the first data point and make a prediction for the second observation, yielding $\text {BF}_{10}(y_2 \mid y_{1}) = p(y_2 \mid y_{1}, \mathcal {H}_{1}) / p(y_2 \mid y_{1}, \mathcal {H}_{0})$ – the relative predictive performance for the second data point, given the knowledge obtained from the first. These one-step-ahead sequential forecasts can be combined –using the law of conditional probability– to produce a model’s overall predictive performance (cf. Dawid’s prequential principle; e.g., Dawid, 1984): BF_10_(*y*) =BF_10_(*y*_1_) ×BF_10_(*y*_2_∣*y*_1_). The accumulation of one-step-ahead sequential forecasts provides a fair assessment of a model’s predictive adequacy, penalizing undue model complexity and thereby implementing a form of Occam’s razor^8^ (i.e., the principle of parsimony, Jefferys & Berger, 1992; Lee & Wagenmakers, 2013; Myung & Pitt, 1997; Myung, Forster, & Browne, 2000; Vandekerckhove, Matzke, & Wagenmakers, 2015; Wagenmakers & Waldorp, 2006). The predictive interpretation of the Bayes factor is conceptually relevant because it means that inference can be meaningful even without either of the models being true in some absolute sense (Morey, Romeijn, & Rouder, 2013; but see van Erven, Grünwald, & de Rooij, 2012).

From the Bayesian perspective, evidence is an inherently relative concept. Therefore it makes little sense to try and evaluate evidence for a specific hypothesis without having specified exactly what the alternative hypothesis predicts. In the words of Peirce (1878a), “When we adopt a certain hypothesis, it is not alone because it will explain the observed facts, but also because the contrary hypothesis would probably lead to results contrary to those observed.” (as quoted in Hartshorne & Weiss, 1932, p. 377). As outlined below, this is one of the main differences with classical hypothesis testing, where the *p* value quantifies the unusualness of the data under the null hypothesis (i.e., the probability of obtaining data at least as extreme as those observed, given that the null hypothesis is true), leaving open the possibility that the data are even more likely under a well-specified and plausible alternative hypothesis.

In sum, Bayes factors compare the predictive adequacy of two competing statistical models. By doing so, they grade the evidence provided by the data on a continuous scale, and quantify the change in belief that the data bring about for the two models under consideration. Its long history and direct link to Bayes’ rule make the Bayes factor “the standard Bayesian solution to the hypothesis testing and model selection problems” (Lewis & Raftery 1997, p. 648) and “the primary tool used in Bayesian inference for hypothesis testing and model selection” (Berger 2006, p. 378). We consider the Bayes factor (or its logarithm) a *thermometer for the intensity of the evidence* (Peirce 1878b). In our opinion, such a thermometer is exactly what researchers desire when they wish to measure the extent to which their observed data support $\mathcal {H}_{1}$ or $\mathcal {H}_{0}$.

### Benefits of Bayesian hypothesis testing

In psychology, several researchers have recently proposed, developed, and promoted Bayes factor hypothesis testing (e.g., Dienes, 2008, 2011, 2014; Hoijtink, 2011; Klugkist et al., 2005; Masson, 2011; Morey & Rouder, 2011; Mulder et al., 2009; Rouder et al., 2009, 2012; Vanpaemel, 2010; Wagenmakers, Lodewyckx, Kuriyal, & Grasman, 2010). Table 1 provides a non-exhaustive list of five specific benefits of Bayesian hypothesis testing over classical *p* value hypothesis testing (see also Kass & Raftery, 1995, p. 773). We now briefly discuss each of these benefits in turn. Other benefits of Bayesian hypothesis testing include those already mentioned for Bayesian parameter estimation above.

#### Benefit 1. The Bayes factor quantifies evidence that the data provide for $\mathcal {H}_{0}$ vs. $\mathcal {H}_{1}$

**Fig. 5:**
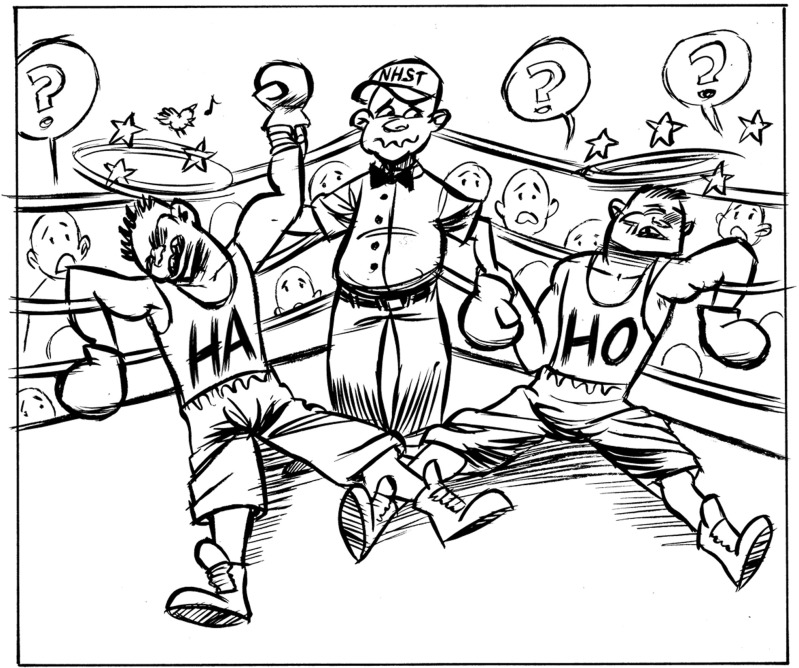
A boxing analogy of the *p* value (Wagenmakers et al., in press). The referee uses null hypothesis significance testing and therefore considers only the deplorable state of boxer $\mathcal {H}_{0}$ (i.e., the null hypothesis). His decision to reject $\mathcal {H}_{0}$ puzzles the public. Figure available at http://www.flickr.com/photos/23868780@N00/12559689854/, courtesy of Dirk-Jan Hoek, under CC license https://creativecommons.org/licenses/by/2.0/

As mentioned above, the Bayes factor is inherently comparative: it weighs the support for one model against that of another. This contrasts with the *p* value, which is calculated conditional on the null hypothesis $\mathcal {H}_{0}$ being true; the alternative hypothesis $\mathcal {H}_{1}$ is left unspecified and hence its predictions are irrelevant as far as the calculation of the *p* value is concerned. Consequently, data that are unlikely under $\mathcal {H}_{0}$ may lead to its rejection, even though these data are just as unlikely under $\mathcal {H}_{1}$ – and are therefore perfectly uninformative (Wagenmakers et al. in press). Figure 5 provides a cartoon highlighting that *p* value NHST considers one side of the coin.

The practical relevance of this concern was underscored by the infamous court case of Sally Clark (Dawid 2005; Hill 2005; Nobles & Schiff 2005). Both of Sally Clark’s children had died at an early age, presumably from cot death or SIDS (sudden infant death syndrome). The probability of a mother having to face such a double tragedy was estimated to be 1 in 73 million. Such a small probability may have influenced judge and jury, who in November 1999 decided to sentence Sally Clark to jail for murdering her two children. In an open letter published in 2002, the president of the Royal Statistical Society Peter Green explained why the probability of 1 in 73 million is meaningless: “The jury needs to weigh up two competing explanations for the babies’ deaths: SIDS or murder. The fact that two deaths by SIDS is quite unlikely is, taken alone, of little value. Two deaths by murder may well be even more unlikely. What matters is the relative likelihood of the deaths under each explanation, not just how unlikely they are under one explanation.” (Nobles & Schiff 2005, p. 19). This point of critique is not just relevant for the case of Sally Clark, but applies to all inferences based on the *p* value.

Bayes factors compare two competing models or hypotheses: $\mathcal {H}_{0}$ and $\mathcal {H}_{1}$. Moreover, Bayes factors do so by fully conditioning on the observed data *y*. In contrast, the *p* value is atail-area integral that depends on hypothetical outcomes more extreme than the one observed in the sample at hand. Such apractice violates the likelihood principle and results in paradoxical conclusions (for examples see Berger & Wolpert, 1988; Wagenmakers, 2007). Indeed, our personal experience suggests that this is one of the most widespread misconceptions that practitioners have about *p* values: interpreting a*p* value as the “probability of obtaining these results given that the null hypothesis is true”. However, as mentioned above, the *p* value equals the probability of obtaining results *at least as extreme* as those observed given that the null hypothesis is true. As remarked by Jeffreys (1980, p. 453): “I have always considered the arguments for the use of Pabsurd. They amount to saying that ahypothesis that may or may not be true is rejected because agreater departure from the trial value was improbable; that is, that it has not predicted something that has not happened.” Towards the end of his life, this critique was acknowledged by one of the main protagonists of the *p* value, Ronald Fisher himself.^9^ In discussing inference for abinomial rate parameter based on observing 3successes out of 14 trials, Fisher argued for the use of likelihood, implicitly acknowledging Jeffreys’s concern:

“Objection has sometimes been made that the method of calculating Confidence Limits by setting an assigned value such as 1% on the frequency of observing 3or less (or at the other end of observing 3or more) is unrealistic in treating the values less than 3, which have not been observed, in exactly the same manner as the value 3, which is the one that has been observed. This feature is indeed not very defensible save as an approximation.” (Fisher 1959, p. 68).

#### Benefit 2. The Bayes factor can quantify evidence in favor of $\mathcal {H}_{0}$

It is evident from Equation 1 that the Bayes factor is able to quantify evidence in favor of $\mathcal {H}_{0}$. In the Bayesian framework, no special status is attached to either of the hypotheses under test; after the models have been specified exactly, the Bayes factor mechanically assesses each model’s one-step-ahead predictive performance, and expresses a preference for the model that was able to make the most accurate series of sequential forecasts (Wagenmakers et al. 2006). When the null hypothesis $\mathcal {H}_{0}$ predicts the observed data better than the alternative hypothesis $\mathcal {H}_{1}$, this signifies that the additional complexity of $\mathcal {H}_{1}$ is not warranted by the data.

The fact that the Bayes factor can quantify evidence in favor of the null hypothesis can be of considerable substantive importance (e.g., Galliset, 2009; Rouder et al., 2009). For instance, the hypothesis of interest may predict an invariance, that is, the absence of an effect across a varying set of conditions. The ability to quantify evidence in favor of the null hypothesis is also important for replication research, and should be of interest to any researcher who wishes to learn whether the observed data provide evidence of absence or absence of evidence (Dienes 2014). Specifically, the possible outcomes of the Bayes factor can be assigned to three discrete categories: (1) evidence in favor of $\mathcal {H}_{1}$ (i.e., evidence in favor of the presence of an effect); (2) evidence in favor of $\mathcal {H}_{0}$ (i.e., evidence in favor of the absence of an effect); (3) evidence that favors neither $\mathcal {H}_{1}$ nor $\mathcal {H}_{0}$. An example of evidence for absence is BF_01_ = 15, where the observed data are 15 times more likely to occur under $\mathcal {H}_{0}$ than under $\mathcal {H}_{1}$. An example of absence of evidence is BF_01_ = 1.5, where the observed data are only 1.5 times more likely to occur under $\mathcal {H}_{0}$ than under $\mathcal {H}_{1}$. Evidentially these scenarios are very different, and it is clearly useful and informative to discriminate between the two. However, the *p* value is not able to make the distinction, and in either of the above scenarios one may obtain *p* = .20. In general, the standard *p* value NHST is unable to provide a measure of evidence in favor of the null hypothesis.

#### Benefit 3. The Bayes factor allows evidence to be monitored as data accumulate

The Bayes factor can be thought of as a thermometer for the intensity of the evidence. This thermometer can be read out, interpreted, and acted on at any point during data collection (cf. the stopping rule principle; Berger & Wolpert, 1988). Using Bayes factors, researchers are free to monitor the evidence as the data come in, and terminate data collection whenever they like, such as when the evidence is deemed sufficiently compelling, or when the researcher has run out of resources (e.g., Berger, 1985, Chapter 7; Edwards et al., 1963; Rouder, 2014; Wagenmaker, 2007). This freedom has substantial practical ramifications, and allows experiments to be conducted in a manner that is both efficient and ethical (e.g., Schönbrodt, Wagenmakers, Zehetleitner, & Perugini, in press).

Consider the hypothetical case where a memory researcher, professor Bumbledorf, has planned to test 40 children with severe epilepsy using intracranial EEG. In scenario 1, Bumbledorf tests 20 children and finds that the data are so compelling that the conclusion hits her straight between the eyes (i.e., Berkson’s interocular traumatic test, Edwards et al., 1963, p. 217). Should Bumbledorf feel forced to test 20 children more, inconveniencing the patients and wasting resources that could be put to better use? In scenario 2, Bumbledorf tests all 40 children and feels that, although the data show a promising trend, the results are not statistically significant (*p* = .11). Should Bumbledorf be disallowed from testing additional children, thereby possibly preventing the patients’ earlier efforts from advancing science by contributing to data that yield an unambiguous conclusion? With Bayes factors, there are no such conundrums (Berger & Mortera 1999); in scenario 1, Bumbledorf can stop after 20 patients and report the Bayes factor; in scenario 2, Bumbledorf is free to continue testing until the results are sufficiently compelling. This freedom stands in sharp contrast to the standard practice of *p* value NHST, where adherence to the sampling plan is critical; this means that according to standard *p* value NHST dogma, Bumbledorf is forced to test the remaining 20 patients in scenario 1 (“why did you even look at the data after 20 patients?”), and Bumbledorf is prohibited from testing addition patients in scenario 2 (“maybe you should have planned for more power”).

It should be acknowledged that the standard framework of *p* value NHST can be adjusted so that it can accommodate sequential testing, either in acontinual fashion, with an undetermined number of tests (e.g., Botella, Ximénez, Revuelta, & Suero, 2006; Fitts, 2010; Frick, 1998; Wald & Wolfowitz, 1948) or in an interrupted fashion, with apredetermined number of tests (e.g., Lakens & Evers, 2014). From aBayesian perspective, however, corrections for sequential monitoring are an anathema. Anscombe (1963, p. 381) summarized the conceptual point of contention:

‘Sequential analysis’ is ahoax(...) So long as all observations are fairly reported, the sequential stopping rule that may or may not have been followed is irrelevant. The experimenter should feel entirely uninhibited about continuing or discontinuing his trial, changing his mind about the stopping rule in the middle, etc., because the interpretation of the observations will be based on what was observed, and not on what might have been observed but wasn’t.

#### Benefit 4. The Bayes factor does not depend on unknown or absent sampling plans

The Bayes factor is not affected by the sampling plan, that is, the intention with which the data were collected. This sampling-plan-irrelevance follows from the likelihood principle (Berger & Wolpert 1988), and it means that Bayes factors may be computed and interpreted even when the intention with which the data are collected is ambiguous, unknown, or absent. This is particularly relevant when the data at hand are obtained from a natural process, and the concepts of “sampling plan” and “experiment” do not apply.

As a concrete demonstration of the practical problems of *p* values when the sampling plan is undefined, consider again the election example and the data shown in Fig. 1. We reported that for this correlation, *p* = .007. However, this *p* value was computed under a fixed sample size scenario; that is, the *p* value was computed under the assumption that an experimenter set out to run 46 elections and then stop. This sampling plan is absurd and by extension, so is the *p* value. But what is the correct sampling plan? It could be something like “US elections will continue every four years until democracy is replaced with a different system of government or the US ceases to exist”. But even this sampling plan is vague – we only learn that we can expect quite a few elections more.

In order to compute a *p* value, one could settle for the fixed sample size scenario and simply not worry about the details of the sampling plan. However, consider the fact that new elections will continue be added to the set. How should such future data be analyzed? One can pretend, after every new election, that the sample size was fixed. However, this myopic perspective induces a multiple comparison problem – every new test has an additional non-zero probability of falsely rejecting the null hypothesis, and the myopic perspective therefore fails to control the overall Type I error rate.^10^

**Fig. 6:**
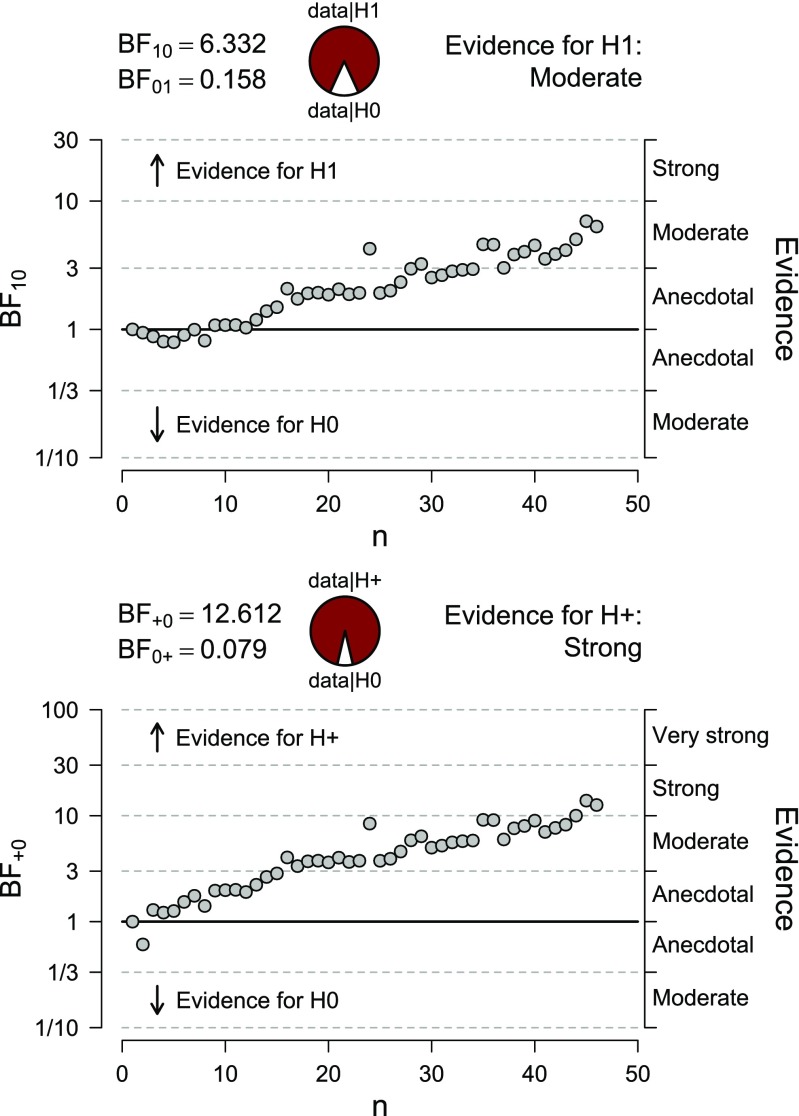
Forthy-six election-long evidential flow for the presence of a correlation between the relative height of the US president and his proportion of the popular vote. *Top panel*: two-sided analysis; *bottom panel*: one-sided analysis. Figure based on JASP

In contrast to *p* value NHST, the Bayes factor can be meaningfully interpreted even when the data at hand have been generated by real-world processes outside of experimental control. Figure 6 shows how the data from the US elections can be analyzed as they come in over time, an updating process that can be extended continually and indefinitely, as long as the US electoral process exists. This example also emphasizes the intimate connection between the benefit of monitoring the evidence as it unfolds over time, and the benefit of being able to compute the evidence from data outside of experimental control: both benefits occur because the Bayes factor does not depend on the intention with which the data are collected (i.e., hypothetical data sets that are not observed).

#### Benefit 5. The Bayes factor is not “violently biased” against $\mathcal {H}_{0}$

Given a complete specification of the models under test, the Bayes factor provides a precise assessment of their relative predictive adequacy. Poor predictive adequacy of $\mathcal {H}_{0}$ alone is not a sufficient reason to prefer $\mathcal {H}_{1}$; it is the balance between predictions from $\mathcal {H}_{0}$ and $\mathcal {H}_{1}$ that is relevant for the assessment of the evidence. As discussed under benefit 1 above, this contrasts with the NHST *p* value, which only considers the unusualness of the data under $\mathcal {H}_{0}$. Consequently, statisticians have repeatedly pointed out that “Classical significance tests are violently biased against the null hypothesis.” (Edwards, 1965, p. 400; see also Johnson, 2013; Sellke et al., 2001). Based on a comparison between *p* values and Bayes factors, Berger & Delampady (1987, p. 330) argued that “First and foremost, when testing precise hypotheses, formal use of P-values should be abandoned. Almost anything will give a better indication of the evidence provided by the data against $\mathcal {H}_{0}$.” In a landmark article, Edwards et al. (1963, p. 228) concluded that “Even the utmost generosity to the alternative hypothesis cannot make the evidence in favor of it as strong as classical significance levels might suggest.” Finally, Lindley suggested, somewhat cynically perhaps, that this bias is precisely the reason for the continued popularity of *p* values: “There is therefore a serious and systematic difference between the Bayesian and Fisherian calculations, in the sense that a Fisherian approach much more easily casts doubt on the null value than does Bayes. Perhaps this is why significance tests are so popular with scientists: they make effects appear so easily.” (Lindley 1986, p. 502).

The *p* value bias against $\mathcal {H}_{0}$ is also evident from the election example, where a correlation of .39, displayed in Fig. 1, yields *p* = .007 and BF_10_ = 6.33. Even though in this particular case both numbers roughly support the same conclusion (i.e., “reject $\mathcal {H}_{0}$” versus “evidence for $\mathcal {H}_{1}$”), the *p* value may suggest that the evidence is compelling, whereas the Bayes factor leaves considerable room for doubt. An extensive empirical comparison between *p* values and Bayes factors can be found in Wetzels et al. (2011). For a Bayesian interpretation of the classical *p* value see Marsman & Wagenmakers (in press).

In sum, the Bayes factor conditions on the observed data to grade the degree of evidence that the data provide for $\mathcal {H}_{0}$ versus $\mathcal {H}_{1}$. As a thermometer for the intensity of the evidence –either for $\mathcal {H}_{0}$ or for $\mathcal {H}_{1}$– the Bayes factor allows researchers to monitor the evidential flow as the data accumulate, and stop whenever they feel the evidence is compelling or the resources have been depleted. Bayes factors can be computed and interpreted even when the intention with which the data have been collected is unknown or entirely absent, such as when the data are provided by a natural process without an experimenter. Moreover, its predictive nature ensures that the Bayes factor does not require either model to be true.

## Ten objections to the Bayes factor hypothesis test

Up to this point we have provided a perspective on Bayesian estimation and Bayesian hypothesis testing that may be perceived as overly optimistic. Bayesian inference does not solve all of the problems that confront the social sciences today. Other important problems include the lack of data sharing and the blurred distinction between exploratory and confirmatory work (e.g., Chambers, 2013; De Groot, 1956/2014; Nosek et al., 2015; Wagenmakers, Wetzels, Borsboom, van der Maas, & Kievit, 2012), not to mention the institutional incentive structure to “publish or perish” (Nosek et al. 2012). Nevertheless, as far as statistical inference is concerned, we believe that the adoption of Bayesian procedures is a definite step in the right direction.

In addition, our enthusiasm for Bayes factor hypothesis testing is shared by only a subset of modern-day Bayesian statisticians (e.g., Albert, 2007; Berger & Pericchi, 2001; Bové & Hekd, 2011; Liang, Paulo, Molina, Clyde, & Berger, 2008; Maruyama & George, 2011; Ntzoufras, Dellaportas, & Forster, 2003; Ntzoufras, 2009; O’Hagan, 1995; Overstall & Forster, 2010; Raftery, 1999; for an alternative perspective see e.g., Robert, 2016). In fact, the topic of Bayes factors is contentious to the extent that it provides a dividing line between different schools of Bayesians. In recognition of this fact, and in order to provide a more balanced presentation, we now discuss a list of ten objections against the approach we have outlined so far. A warning to the uninitiated reader: some of the objections and counterarguments may be difficult to understand from a superficial reading alone; trained statisticians and philosophers have debated these issues for many decades, without much resolution in sight.

### Objection 1: Estimation is always superior to testing

As mentioned in the introduction, it is sometimes argued that researchers should abandon hypothesis tests in favor of parameter estimation (e.g., Cumming, 2014). We agree that parameter estimation is an important and unduly neglected part of the inductive process in current-day experimental psychology, but we believe that ultimately both hypothesis testing and parameter estimation have their place, and a complete report features results from both approaches (Berger 2006).

Parameter estimation is most appropriate when the null hypothesis is not of any substantive research interest. For instance, in political science one may be interested in polls that measure the relative popularity of various electoral candidates; the hypothesis that all candidates are equally popular is uninteresting and irrelevant. Parameter estimation is also appropriate when earlier work has conclusively ruled out the null hypothesis as a reasonable explanation of the phenomenon under consideration. For instance, a study of the Stroop effect need not assign prior mass to the hypothesis that the effect is absent. In sum, whenever prior knowledge or practical considerations rule out the null hypothesis as a plausible or interesting explanation then a parameter estimation approach is entirely defensible and appropriate.

Other research scenarios, however, present legitimate testing problems. An extreme example concerns precognition: the question at hand is not “Assuming that people can look into the future, how strong is the effect?” – rather, the pertinent question is “Can people look into the future?”. The same holds for medical clinical trials, where the question at hand is not “Assuming the new treatment works, how strong is the effect?” but instead is “Does the new treatment work?”. Note that in these examples, the parameter estimation question presupposes that the effect exists, whereas the hypothesis testing question addresses whether that supposition is warranted in the first place.

The relation between estimation and testing is discussed in detail in Jeffreys’s book “Theory of Probability”. For instance, Jeffreys provides aconcrete example of the difference between estimation and testing:

“The distinction between problems of estimation and significance arises in biological applications, though Ihave naturally tended to speak mainly of physical ones. Suppose that aMendelian finds in abreeding experiment 459 members of one type, 137 of the other. The expectations on the basis of a3 : 1 ratio would be 447 and 149. The difference would be declared not significant by any test. But the attitude that refuses to attach any meaning to the statement that the simple rule is right must apparently say that if any predictions are to be made from the observations the best that can be done is to make them on the basis of the ratio 459/137, with allowance for the uncertainty of sampling. Isay that the best is to use the 3/1 rule, considering no uncertainty beyond the sampling errors of the new experiments. In fact the latter is what ageneticist would do. The observed result would be recorded and might possibly be reconsidered at alater stage if there was some question of differences of viability after many more observations had accumulated; but meanwhile it would be regarded as confirmation of the theoretical value. This is aproblem of what Icall significance.

But what are called significance tests in agricultural experiments seem to me to be very largely problems of pure estimation. When aset of varieties of aplant are tested for productiveness, or when various treatments are tested, it does not appear to me that the question of presence or absence of differences comes into consideration at all. It is already known that varieties habitually differ and that treatments have different effects, and the problem is to decide which is the best; that is, to put the various members, as far as possible, in their correct order.” (Jeffreys 1961, p. 389).^11^

Moreover, Jeffreys argues that asole reliance on estimation results in inferential chaos:

“These are all problems of pure estimation. But their use as significance tests covers alooseness of statement of what question is being asked. They give the correct answer if the question is: If there is nothing to require consideration of some special values of the parameter, what is the probability distribution of that parameter given the observations? But the question that concerns us in significance tests is: If some special value has to be excluded before we can assert any other value, what is the best rule, on the data available, for deciding whether to retain it or adopt anew one? The former is what Icall aproblem of estimation, the latter of significance. Some feeling of discomfort seems to attach itself to the assertion of the special value as *right* since it may be slightly wrong but not sufficiently to be revealed by atest on the data available; but no significance test asserts it as certainly right. We are aiming at the best way of progress, not at the unattainable ideal of immediate certainty. What happens if the null hypothesis is retained after asignificance test is that the maximum likelihood solution or asolution given by some other method of estimation is rejected. The question is, When we do this, do we expect thereby to get more or less correct inferences than if we followed the rule of keeping the estimation solution regardless of any question of significance? Imaintain that the only possible answer is that we expect to get more. The difference as estimated is interpreted as random error and irrelevant to future observations. In the last resort, if this interpretation is rejected, there is no escape from the admission that anew parameter may be needed for every observation, and then all combination of observations is meaningless, and the only valid presentation of data is amere catalogue without any summaries at all.” (Jeffreys 1961, pp. 387–388)

In light of these and other remarks, Jeffreys’s maxim may be stated as follows: “Do not try to estimate something until you are sure there is something to be estimated.”^12^

Finally, in some applications the question of estimation never arises. Examples include cryptography (Turing 1941/2012; Zabell 2012), the construction of phylogenetic trees (Huelsenbeck & Ronquist 2001), and the comparison of structurally different models (e.g., in the field of response time analysis: the diffusion model versus the linear ballistic accumulator model; in the field of categorization: prototype versus exemplar models; in the field of visual working memory: discrete slot models versus continuous resource models; in the field of long-term memory: multinomial processing tree models versus models based on signal detection theory).

In sum, hypothesis testing and parameter estimation are both important. In the early stages of a research paradigm, the focus of interest may be on whether the effect is present or absent; in the later stages, if the presence of the effect has been firmly established, the focus may shift towards an estimation approach.

### Objection 2: Bayesian hypothesis tests can indicate evidence for small effects that are practically meaningless

An objection that is often raised against NHST may also be raised against Bayes factor hypothesis testing: with large sample sizes, even small and practically meaningless effects will be deemed “significant” or “strongly supported by the data”. This is true. However, what is practically relevant is context-dependent – in some contexts, small effects can have large consequences. For example, Goldstein, Cialdini, and Griskevicius (2008) reported that messages to promote hotel towel reuse are more effective when they also attend guests to descriptive norms (e.g., “the majority of guests reuse their towels”). Based on a total of seven published experiments, a Bayesian meta-analysis suggests that this effect is present (BF_10_ ≈ 37) but relatively small, around 6% (Scheibehenne, Jamil, & Wagenmakers, in press). The practical relevance of this result depends on whether or not it changes hotel policy; the decision to change the messages or leave them intact requires hotels to weigh the costs of changing the messages against the expected gains from having to wash fewer towels; for a large hotel, a 6% gain may result in considerable savings.

Thus, from a Bayesian perspective, context-dependence is recognized and incorporated through an analysis that computes expected utilities for a set of possible actions (Lindley 1985). The best action is the one with the highest expected utility. In other words, the practicality of the effects can be taken into account, if needed, by adding an additional layer of considerations concerning utility. Another method to address this objection is to specify the null hypothesis not as a point but as a practically relevant interval around zero (Morey & Rouder 2011).^13^

### Objection 3: Bayesian hypothesis tests promote binary decisions

It is true that Jeffreys and other statisticians have suggested rough descriptive guidelines for the Bayes factor (for a more detailed discussion see Wagenmakers et al., this issue). These guidelines facilitate a discrete verbal summary of a quantity that is inherently continuous. More importantly, regardless of whether it is presented in continuous numerical or discrete verbal form, the Bayes factor grades the evidence that the data provide for $\mathcal {H}_{0}$ versus $\mathcal {H}_{1}$ – thus, the Bayes factor relates to evidence, not decisions (Ly, Verhagen, & Wagenmakers, 2016a). As pointed out above, decisions require a consideration of actions and utilities of outcomes (Lindley 1985). In other words, the Bayes factor measure the change in beliefs brought about by the data, or –alternatively– the relative predictive adequacy of two competing models; in contrast, decisions involve the additional consideration of actions and their consequences.

### Objection 4: Bayesian hypothesis tests are meaningless under misspecification

The Bayes factor is a measure of relative rather than absolute performance. When the Bayes factor indicates overwhelming support in favor of $\mathcal {H}_{1}$ over $\mathcal {H}_{0}$, for instance, this does not imply that $\mathcal {H}_{1}$ provides an acceptable account of the data. Instead, the Bayes factor indicates only that the predictive performance of $\mathcal {H}_{1}$ is superior to that of $\mathcal {H}_{0}$; the absolute performance of $\mathcal {H}_{1}$ may well be abysmal.

A simple example illustrates the point. Consider a test for a binomial proportion parameter *𝜃*. Assume that the null hypothesis specifies a value of interest *𝜃*_0_, and assume that the alternative hypothesis postulates that *𝜃* is lower than *𝜃*_0_, with each value of *𝜃* judged equally likely a priori. Hence, the Bayes factor compares $\mathcal {H}_{0}: \theta = \theta _{0}$ against $\mathcal {H}_{1}: \theta \sim \text {Uniform}(0,\theta _{0})$ (e.g., Haldane, 1932; Etz & Wagenmakers, 2016). Now assume that the data consist of a sequence of length *n* that features only successes (e.g., items answered correctly, coin tosses landing tails, patients being cured). In this case the predictions of $\mathcal {H}_{0}$ are superior to those of $\mathcal {H}_{1}$. A straightforward derivation^14^ shows that the Bayes factor in favor of $\mathcal {H}_{0}$ against $\mathcal {H}_{1}$ equals *n* + 1, *regardless* of *𝜃*_0_.^15^ Thus, when *n* is large the Bayes factor will indicate decisive relative support in favor of $\mathcal {H}_{0}$ over $\mathcal {H}_{1}$; at the same time, however, the absolute predictive performance of $\mathcal {H}_{0}$ depends crucially on *𝜃*_0_, and becomes abysmal when *𝜃*_0_ is low.

**Fig. 7:**
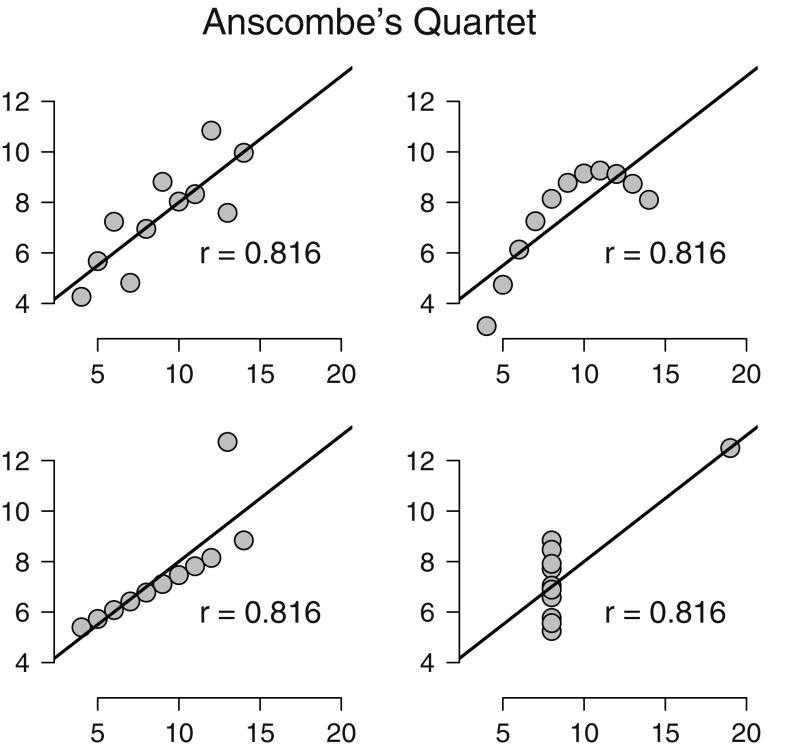
“Anscombe’s quartet highlights the importance of plotting data to confirm the validity of the model fit. In each panel, the Pearson correlation between the *x* and *y* values is the same, *r* = 0.816. In fact, the four different data sets are also equal in terms of the mean and variance of the *x* and *y* values. Despite the equivalence of the four data patterns in terms of popular summary measures, the graphical displays reveal that the patterns are very different from one another, and that the Pearson correlation (a linear measure of association) is only valid for the data set from the top left panel.” (Heathcote et al. 2015, p. 34). Figure available at http://tinyurl.com/zv2shlx under CC license https://creativecommons.org/licenses/by/2.0/

The critique that the Bayes factor does not quantify absolute fit is therefore entirely correct, but it pertains to statistical modeling across the board. Before drawing strong inferential conclusions, it is always wise to plot the data, inspect residuals, and generally confirm that the model under consideration is not misspecified in a major way. The canonical example of this is Anscombe’s quartet, displayed here in Fig. 7 (see also Andraszewics et al., 2015; Anscombe, 1973; Heathcote, Brown, & Wagenmakers, 2015; Lindsay, 2015). Each panel of the quartet displays two variables with the same mean and variance. Moreover, for the data in each panel the Pearson correlation coefficient equals *r* = 0.816. An automatic analysis of the data from each panel yields the same four *p* values, the same four confidence intervals, the same four Bayes factors, and the same four credible intervals. Yet a mere glance at Fig. 7 suggests that these inferential conclusions are meaningful only for the data from the top left panel.

### Objection 5: vague priors are preferable over informed priors

Bayes factors cannot be used with extremely vague or “uninformative” prior distributions for the parameters under test. For instance, a *t*-test on effect size *δ* cannot specify $\mathcal {H}_{1}: \delta \sim \text {Uniform}(-\infty , \infty )$, as this leaves the Bayes factor undefined. The use of an almost uninformative prior does not solve the problem; the specification $\mathcal {H}_{1}: \delta \sim \text {Uniform}(-10^{100}, 10^{100})$ means that for all sets of reasonable data, the null hypothesis will be strongly preferred. The reason for this behavior is that with such a vague prior, $\mathcal {H}_{1}$ predicts that effect size is virtually certain to be enormous; these predictions are absurd, and $\mathcal {H}_{1}$ is punished accordingly (Rouder & Morey 2012).

Consequently, a reasonable comparison between $\mathcal {H}_{0}$ and $\mathcal {H}_{1}$ requires that both models are specified in a reasonable way (e.g., Dienes, 2011; Vanpaemel, 2010; Vanpaemel & Lee, 2012). Vague priors for effect size are not reasonable. In parameter estimation such unreasonableness usually does not have negative consequences, but this is different for Bayes factor hypothesis testing. Thus, the core problem is not with Bayes factors – the core problem is with unreasonable prior distributions.

### Objection 6: default priors are not sufficiently subjective

Jeffreys (1961) and other “objective” Bayesians have proposed default priors that are intended to be used regardless of the area of substantive application. These default priors provide a reference result that can be refined by including subjective knowledge. However, “subjective” Bayesians may argue that this needs to be done always, and the subjectivity in the specification of priors for Bayes factor hypothesis testing does not go far enough. For instance, the *t*-test involves the specification $\mathcal {H}_{1}: \delta \sim \text {Cauchy}(0,r)$. But is it reasonable for the Cauchy distribution to be centered on zero, such that the most likely value for effect size under $\mathcal {H}_{1}$ equals zero? Perhaps not (e.g., Johnson, 2013). In addition, the Cauchy form itself may be questioned. Perhaps each analysis attempt should be preceded by a detailed prior elicitation process, such that $\mathcal {H}_{1}$ can be specified in a manner that incorporates all prior knowledge that can be brought to bear on the problem at hand.

The philosophical position of the subjective Bayesian is unassailable, and if the stakes are high enough then every researcher would do well to turn into a subjective Bayesian. However, the objective or consensus Bayesian methodology affords substantial practical advantages: it requires less effort, less knowledge, and it facilitates communication (e.g., Berger, 2004; but see Goldstein, 2006). For more complicated models, it is difficult to see how a subjective specification can be achieved in finite time. Moreover, the results of an objective analysis may be more compelling to other researchers than those of a subjective analysis (Morey, Wagenmakers, & Rouder, in press). Finally, in our experience, the default priors usually yield results that are broadly consistent with those that would be obtained with a more subjective analysis (see also Jeffreys, 1963). Nevertheless, the exploration of more subjective specifications requires more attention (e.g., Dienes, 2014; Verhagen & Wagenmakers, 2014).

### Objection 7: subjective priors are not sufficiently objective

This is an often-heard objection to Bayesian inference in general: the priors are subjective, and in scientific communication one needs to avoid subjectivity at all cost. Of course, this objection ignores the fact that the specification of statistical models is also subjective – the choice between probit regression, logistic regression, and hierarchical zero-inflated Poisson regression is motivated subjectively, by a mix of prior knowledge and experience with the statistical model under consideration. The same holds for power analyses that are conducted using a particular effect size, the choice of which is based on a subjective combination of previous experimental outcomes and prior knowledge. Moreover, the scientific choices of what hypothesis to test, and how to design a good experiment are all subjective. Despite their subjectivity, the research community has been able, by and large, to assess the reasonableness of the choices made by individual researchers.

When the choice is between a method that is objective but unreasonable versus a method that is subjective but reasonable, most researchers would prefer the latter. The default priors for the Bayes factor hypothesis tests are a compromise solution: they attempt to be reasonable without requiring a complete subjective specification.

### Objection 8: default priors are prejudiced against small effects

On his influential blog, Simonsohn has recently argued that default Bayes factor hypothesis tests are prejudiced against small effects.^16^ This claim raises the question “Prejudiced compared to what?”. Small effects certainly receive more support from a classical analysis, but, as discussed above, this occurs mainly because the classical paradigm is biased against the null as the predictions made by $\mathcal {H}_{1}$ are ignored (cf. Fig. 5). Furthermore, note that for large sample sizes, Bayes factors are guaranteed to strongly support a true $\mathcal {H}_{1}$, even for very small true effect sizes. Moreover, the default nested prior specification of $\mathcal {H}_{1}$ makes it difficult to collect compelling evidence for $\mathcal {H}_{0}$, so the most prominent advantage is generally with $\mathcal {H}_{1}$, not with $\mathcal {H}_{0}$.

These considerations mean that a Bayes factor analysis may be misleading only under the following combination of factors: a small sample size, a small true effect size, and a prior distribution that represents the expectation that effect size is large. Even under this unfortunate combination of circumstances, the extent to which the evidence is misleading will be modest, at least for reasonable prior distributions and reasonable true effect sizes. The relevant comparison is not between the default Bayes factor and some unattainable Platonic ideal; the relevant comparison is between default Bayes factors and *p* values. Here we believe that practical experience will show that Bayes factors are more informative and have higher predictive success than that provided by *p* values.

### Objection 9: increasing sample size solves all statistical problems

An increase in sample size will generally reduce the need for statistical inference: with large samples, the signal-to-noise ratio often becomes so high that the data pass Berkson’s interocular traumatic test. However, “The interocular traumatic test is simple, commands general agreement, and is often applicable; well-conducted experiments often come out that way. But the enthusiast’s interocular trauma may be the skeptic’s random error. A little arithmetic to verify the extent of the trauma can yield great peace of mind for little cost.” (Edwards et al. 1963, p. 217).

Moreover, even high-powered experiments can yield completely uninformative results (Wagenmakers, Verhagen, & Ly, 2016). Consider Study 6 from Donnellan, Lucas, and Cesario (2015), one of nine replication attempts on the reported phenomenon that lonely people take hotter showers (in order to replace the lack of social warmth with physical warmth; Bargh & Shalev, 2012). Although the overall results provided compelling evidence in favor of the null hypothesis (Wagenmakers, Verhagen, & Ly, 2016), three of the nine studies by Donnellan et al. (2015) produced only weak evidence for $\mathcal {H}_{0}$, despite relatively large sample sizes. For instance, Study 6 featured *n* = 553 with *r* = .08, yielding a one-sided *p* = 0.03. However, the default one-sided Bayes factor equals an almost perfectly uninformative BF_0+_ = 1.61. This example demonstrates that a high-powered experiment does not need to provide diagnostic information; power is a pre-experimental concept that is obtained by considering all the hypothetical data sets that can be observed. In contrast, evidence is a post-experimental concept, taking into account only the data set that was actually obtained (Wagenmakers et al. 2015).

### Objection 10: Bayesian procedures can be hacked too

In an unpublished paper, Simonsohn has argued that Bayes factors are not immune to the biasing effects of selective reporting, ad-hoc use of transformations and outlier removal, etc. (Simonsohn 2015a).^17^ In other words, Bayes factors can be “hacked” too, just like *p* values. This observation is of course entirely correct. Any reasonable statistical method should be sensitive to selective reporting, for else it does not draw the correct conclusions in case the data were obtained without it. Bayes factors are elegant and often informative, but they cannot work miracles and the value of a Bayes factor rests on the reliability and representativeness of the data at hand.

The following example illustrates a more subtle case of “B-hacking” that is able to skew statistical conclusions obtained from a series of experiments. In 2011, Bem published an article in the *Journal of Personality and Social Psychology* in which he argued that eight of nine experiments provided statistical evidence for precognition (Bem 2011), that is, the ability of people to anticipate a completely random event (e.g., on which side of the computer screen a picture is going to appear). A default Bayes factor analysis by Wagenmakers, Wetzels, Borsboom, and van der Maas (2011) showed that the evidence was not compelling and in many cases even supported $\mathcal {H}_{0}$. In response, Bem, Utts, and Johnson (2011) critiqued the default prior distribution and re-analyzed the data using their own subjective “precognition prior”. Based on this prior distribution, Bem et al. (2011) reported a combined Bayes factor of 13,669 in favor of $\mathcal {H}_{1}$. The results seems to contrast starkly with those of Wagenmakers et al. (2011); can the subjective specification of the prior distribution exert such a huge effect?

The conflict between Bem et al. (2011) and Wagenmakers et al. (2011) is more apparent than real. For each experiment separately, the Bayes factors from Bem et al. (2011) and Wagenmakers et al. (2011) are relatively similar, a result anticipated by the sensitivity analysis reported in the online supplement to Wagenmakers et al. (2011). The impressive Bayes factor of 13,669 in favor of the precognition hypothesis was obtained by multiplying the Bayes factors for the individual experiments. However, this changes the focus of inference from individual studies to the entire collection of studies as a whole. Moreover, as explained above, multiplying Bayes factors without updating the prior distribution is a statistical mistake (Jeffreys 1961; Rouder & Morey 2011; Wagenmakers et al. 2016).

In sum, the Bayes factor conclusions from Bem et al. (2011) and Wagenmakers et al. (2011) are in qualitative agreement about the relatively low evidential impact of the individual studies reported in Bem (2011). The impression of a conflict is caused by a change in inferential focus coupled with a statistical mistake. Bayesian inference is coherent and optimal, but it is not a magic potion that protects against malice or statistical misunderstanding.

## Concluding comments

Substantial practical rewards await the pragmatic researcher who decides to adopt Bayesian methods of parameter estimation and hypothesis testing. Bayesian methods can incorporate prior information, they do not depend on the intention with which the data were collected, and they can be used to quantify and monitor evidence, both in favor of $\mathcal {H}_{0}$ and $\mathcal {H}_{1}$. In depressing contrast, classical procedures apply only in the complete absence of knowledge about the topic at hand, they require knowledge of the intention with which the data were collected, they are biased against the null hypothesis, and they can yield conclusions that, although valid on average, may be absurd for the case at hand.

Despite the epistemological richness and practical benefits of Bayesian parameter estimation and Bayesian hypothesis testing, the practice of reporting *p* values continues its dominant reign. As outlined in the introduction, the reasons for resisting statistical innovation are manyfold (Sharpe 2013). In recent years our work has focused on overcoming one reason for resistance: the real or perceived difficulty of obtaining default Bayesian answers for run-of-the-mill statistical scenarios involving correlations, the *t*-test, ANOVA and others. To this aim we have developed JASP, a software program that allows the user to conduct both classical and Bayesian analyses.^18^ An in-depth discussion of JASP is provided in Part II of this series (Wagenmakers et al. this issue).

## Footnotes

1 These arguments are speculative to the degree that they are based entirely on our personal experience and common-sense; in other words, our arguments have not been subjected to rigorous empirical tests.

2 The prior distributions for the other parameters from the bivariate Normal are inconsequential for inference about *ρ* and can be assigned vague prior distributions (Ly et al. 2016b). A slightly different and less transparent Bayesian model for the Pearson correlation coefficient is presented in Wetzels & Wagenmakers (2012).

3 The rationale behind the one-sided classical confidence interval is difficult to teach. One statistics teacher remarked “one-sided classical confidence intervals really blow students’ minds, and not in a good way.” Another statistics teacher said that she simply refuses to cover the concept at all, in order to prevent student riots.

4 This difference was already clear to Laplace, who argued that the post-data viewpoint is “obviously” the one that should be employed (Gillispie 1997, p. 82).

5 Using maximum likelihood estimation, general-purpose gradient decent algorithms in Matlab, R, and Excel often fail in nonlinear contexts with more than just a few dozen parameters.

6 Under a continuous prior probability distribution, the probability assigned to any single point (i.e., *ρ* = 0) is zero.

7 Specification of prior distributions is an important component for Bayes factor hypothesis testing, as the prior distributions define a model’s complexity and hence exert a lasting effect on the test outcome. We will return to this issue later.

8 An overly complex model mistakes noise for signal, tailoring its parameters to data patterns that are idiosyncratic and nonrepeatable. This predilection to “overfit” is exposed when the model is forced to make out-of-sample predictions, because such predictions will be based partly on noise.

9 The first *p* value was calculated by Pierre-Simon Laplace in the 1770s; the concept was formally introduced by Karl Pearson in 1900 as a central component to his Chi-squared test (http://en.wikipedia.org/wiki/P-value#History).

10 For sequential tests the multiple comparisons are not independent; this reduces but does not eliminate the rate with which the Type I error increases.

11 Jeffreys’s statement that treatment effects are the domain of estimation may appear inconsistent with our claim that medical clinical trials are the domain of testing. However, the difference is that Jeffreys’s treatment effects are random, whereas the treatment in aclinical trial is targeted (see also footnote 1in Bayarri, Benjamin, Berger, & Sellke, 2016).

12 This is inspired by what is known as Hyman’s maxim for ESP, namely “Do not try to explain something until you are sure there is something to be explained.” (Alcock, 1994, p. 189, see also http://www.skeptic.com/insight/history-and-hymans-maxim-part-one/). For a similar perspective see Paul Alper’s comment on what Harriet Hall termed “Tooth fairy science” https://www.causeweb.org/wiki/chance/index.php/Chance_News_104#Tooth_fairy_science: “Yes, you have learned something. But you haven’t learned what you think you’ve learned, because you haven’t bothered to establish whether the Tooth Fairy really exists”.

13 We plan to include this functionality in a future version of JASP.

14 See supplemental materials available at the Open Science Framework, https://osf.io/m6bi8/.

15 This surprising result holds as long as *𝜃*_0_ > 0.

16
http://datacolada.org/2015/04/09/35-the-default-bayesian-test-is-prejudiced-against-small-effects/ http://datacolada.org/2015/04/09/35-the-default-bayesian-test-is-prejudiced-against-small-effects/

17 The paper is available at http://papers.ssrn.com/sol3/papers.cfm?abstract_id=2374040.

18 The development of JASP was made possible by the ERC grant “Bayes or bust: Sensible hypothesis tests for social scientists”.
